# Non-Destructive Sensor-Based Prediction of Maturity and Optimum Harvest Date of Sweet Cherry Fruit

**DOI:** 10.3390/s17020277

**Published:** 2017-01-31

**Authors:** Verena Overbeck, Michaela Schmitz, Michael Blanke

**Affiliations:** 1 INRES-Horticultural Science, Faculty of Agriculture, University of Bonn, D-53121 Bonn, Germany; v.overbeck@gmx.de; 2 Department of Applied Science, Bonn-Rhein-Sieg University, D-53359 Rheinbach, Germany; Michaela.Schmitz@h-brs.de

**Keywords:** Sweet cherry (*Prunus avium* L.), bio-innovation, harvest prediction, maturity index, modeling, NAI, NDVI, nondestructive examination

## Abstract

(1) Background: The aim of the study was to use innovative sensor technology for non-destructive determination and prediction of optimum harvest date (OHD), using sweet cherry as a model fruit, based on different ripening parameters. (2) Methods: Two cherry varieties in two growing systems viz. field and polytunnel in two years were employed. The fruit quality parameters such as fruit weight and size proved unsuitable to detect OHD alone due to their dependence on crop load, climatic conditions, cultural practices, and season. Coloration during cherry ripening was characterized by a complete decline of green chlorophyll and saturation of the red anthocyanins, and was measured with a portable sensor viz. spectrometer 3–4 weeks before expected harvest until 2 weeks after harvest. (3) Results: Expressed as green NDVI (normalized differential vegetation index) and red NAI (normalized anthocyanin index) values, NAI increased from −0.5 (unripe) to +0.7 to +0.8 in mature fruit and remained at this saturation level with overripe fruits, irrespective of variety, treatment, and year. A model was developed to predict the OHD, which coincided with when NDVI reached and exceeded zero and the first derivative of NAI asymptotically approached zero. (4) Conclusion: The use of this sensor technology appears suitable for several cherry varieties and growing systems to predict the optimum harvest date.

## 1. Introduction

All four maturity indices for the determination of the optimum harvest date (OHD) for pome fruit, such as (1) Streif index; (2) Perlim index; (3) Thiault index [1]; and (4) De Jager index [2,3,4], include starch and are destructive. These maturity indices, however, are lacking for stone fruit—such as plum, peach, nectarine, apricot, and cherry—because starch is scarce in these fruits [5] and the changes are too small for application of a Streif or other index. The harvest date influences the content of secondary compounds such as phenolic ingredients and pigments; these bioactive compounds enhance the nutritional and health value of a fruit, and additionally function to visually attract consumers in terms of fruit coloration. Current evidence strongly supports the role of polyphenols in the prevention of cardiovascular diseases, cancers, arteriosclerosis, and other age-related diseases [6]. Other maturity parameters such as fruit size, flesh firmness, and concentration of soluble solids are affected by crop load on the tree, climatic conditions, cultural practices, and season and are therefore less suitable as a maturity index alone [7]. Correlation between spectral measurements and ripening parameters showed that spectral measurements are useful to non-destructively determine changes in fruit color [8,9], acting as a potential ripeness indicator and offering the earliest opportunity to obtain information about the optimum harvest date [10]. De Jager and Roelofs [2] used the a* value in the CIE L*a*b* color scheme to improve the Streif index. Portable colorimeters can be alternatives to spectrometric measurements of the visible wavelengths, but the results of the measurements are difficult for the user to interpret. At the same time, the a* values, which relate to the red–green distinction of human vision, poorly describe the nonlinear and seasonal variation of pigment content [11,12].

Overall, maturation and ripening in most fruits are associated with a decrease in chlorophyll content, which drops to near-zero values in plum and cherry fruit, thereby excluding the use of chlorophyll fluorescence as a non-destructive method for maturity assessment in these stone fruits.

In pome fruit (e.g., apple) the background color, which is related to the chlorophyll content, can be used to follow the maturation [13] and to determine the optimum harvest date. Zude-Sasse et al. [12] showed that the optical determination of the decline in chlorophyll content is a promising tool to determine the optimal harvest date of apple fruit. This decrease in chlorophyll content combined with an increase in carotenoid content can be considered as an indicator of the stage of fruit ripening and of fruit quality [8,9]. Modern biotechnology (e.g., sensors) can measure these changes in pigment contents in a nondestructive way, so that such measurements can be used in situ by producers and extension services in an inexpensive and easy way [7]. The objective of the present work was to investigate the sensor technology as non-destructive, portable method with a modification of existing affordable equipment to determine changes in ripening parameters, using sweet cherry as a model for stone fruit.

## 2. Materials and Methods

### 2.1. Plant Material

Four-year-old sweet cherry (*Prunus avium* L.) trees of cultivars “Samba” and “Bellise” on dwarfing Gisela 3 rootstock were grown at a spacing of 2.70 × 2.00 m at Campus Klein-Altendorf (latitude 50.5° N), University of Bonn, Germany. Cherry trees were either cultivated in the field or in polytunnels (Haygrove Ltd., Ledbury, Herefordshire, U.K.) with a Visqueen luminance plastic cover to increase diffuse light and to force cherries to ripen 2 weeks earlier than the uncovered trees outside and to create variability in ripening patterns for the sensor application.

### 2.2. Instrumentation and Sampling

Cherry fruits were examined with a plant sensor (portable spectrometer) at 2-day intervals from 3–4 weeks before anticipated harvest at the onset of color change (breaker stage) until 2 weeks after commercial harvesting to cover all maturation stages.

A photodiode array spectrometer (type pigment analyzer 1101, Control in Applied Physiology, Berlin-Falkensee, Germany) was used to non-destructively measure relative changes in concentration of chlorophyll and anthocyanins on two opposite sides of the cherry fruits. A total of 30 fruits from four trees of every cultivar and treatment were used from the lower part of the tree crown for an optimum composite sample, resulting in 120 determinations on each sampling date.

**Figure 1:**
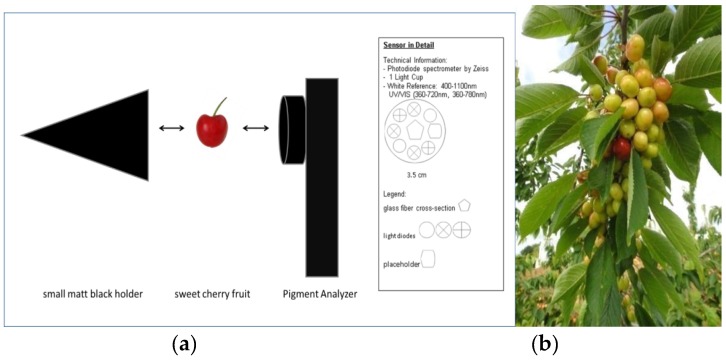
Sensor technology (**a**) to non-destructively determine normalized anthocyanin index (NAI) and normalized differential vegetation index (NDVI) of sweet cherry fruit; an existing PA (Pigment Analyzer type 1101) was modified; a matt black holder was built for the device to reduce stray light and (**b**) “breaker stage” of the cherry fruit from green to yellow, measurements should begin before this stage in the proposed method.

The head of the instrument contains five light-emitting diodes (LEDs) as light-emitting sources in a ring and the center detector (Figure 1a), which measures the light spectrum in the range of 400–1100 nm remitted from the fruit peel. Relative changes in chlorophyll concentration are expressed as normalized differential vegetation index or NDVI = (I780 − I660)/(I780 + I660) and anthocyanins as normalized anthocyanins index or NAI = (I780 − I570)/(I780 + I570) with a disposition of both parameters normalized to between −1 (lack of green or redness) and +1 (green or red) [14]. The spectrometer was originally built for cv. “Elstar” apple fruits (i.e., not for cherries or other fruits smaller than apples). Hence, we machined a small matt black holder for the cherries to exclude any stray light from the spectrophotometer light detector (Figure 1).

### 2.3. Fruit Quality Assessment

Starting at the breaker stage, when the fruit start to turn yellow, all fruit quality attributes were assessed for 30 cherry fruits per treatment and cultivar every 3 days during maturation for both varieties, treatments, and years. Sugar, acidity, and color were determined using standard procedures [15]. Sugar was measured as total soluble solids (TSS) using a digital refractometer type PR 32 (Atago Co., Tokyo, Japan). Fruit firmness was measured on both sides of the cherry fruit equator with a Bareiss penetrometer with a 2 mm plunger; fruit diameter was measured with a caliper and the weight of the fruits, including the stalk, was determined with a digital balance. Organoleptic tests were carried out to support the chemical analysis (data not shown).

### 2.4. Determination of Secondary Compounds

#### 2.4.1. Preparation of Cherry Fruits for Measurements

Sweet cherry (*Prunus avium* L.) fruits picked from the lower part of the tree without visual skin defects were used first for quality assessment and afterwards for the chemical analysis. The cherry fruit were sampled twice a week from the onset of fruit maturation: the stone was removed, the fruit macerated, and then the mash frozen at −80 °C until the chemical pigment analysis [16].

#### 2.4.2. Pigment Analysis

The extraction procedure allowed the simultaneous assay of chlorophylls, carotenoids, flavonoids (such as quercetin glycosides), and anthocyanins in an extract (Solovchenko and Schmitz-Eiberger) [16]. The fruit mash was homogenized in chloroform/methanol (2/1, *v*/*v*) in the presence of MgO and filtered through paper. Distilled water (1/5 of total extract volume) was added and extracts were centrifuged at 3000× *g* for 10 min until phase separation. Absorbance of the extracts was measured with a spectrophotometer, type Lambda 15 (Bodenseewerk Perkin-Elmer GmbH & Co. KG, Überlingen, Germany). Chlorophyll and carotenoid concentrations were quantified in the lower (chloroform) phase using Wellburn’s [17] coefficients, and the upper (water ± methanol) phase of the extract was used for measuring flavonoids and anthocyanins. Pigment content was expressed based on the weight of fruit mash.

### 2.5. Statistics

Experimental data were analyzed using the statistical program SPSS 21.0 (SPSS Inc., Chicago, IL, USA). For all fruit quality parameters, the Kolmogorov–Smirnov test was used to assess the data for normal distribution. All quality parameters and the NDVI and NAI were normally distributed.

## 3. Results

### 3.1. Mircroclimate

The incident light was measured diurnally at a height of 2 m from the ground on 26 June 2012 (solar angle 63°). Photosynthetically active radiation (PAR = maximum 2118 µmol PAR m^−2^·s^−1^ at 12 a.m.; minimum 500 µmol PAR m^−2^·s^−1^ at 6 p.m.) was reduced in the polytunnel by up to 34% in the morning (10 a.m.) and by a maximum of 45% in the evening (6 p.m.) (result not shown).

### 3.2. Fruit Quality

Size and weight increased with fruit ontogeny in all treatments and years from 16 mm fruit size “Bellise without cover” and increased to 26 mm in the treatment “Samba with cover” (Table 1).

**Figure 2:**
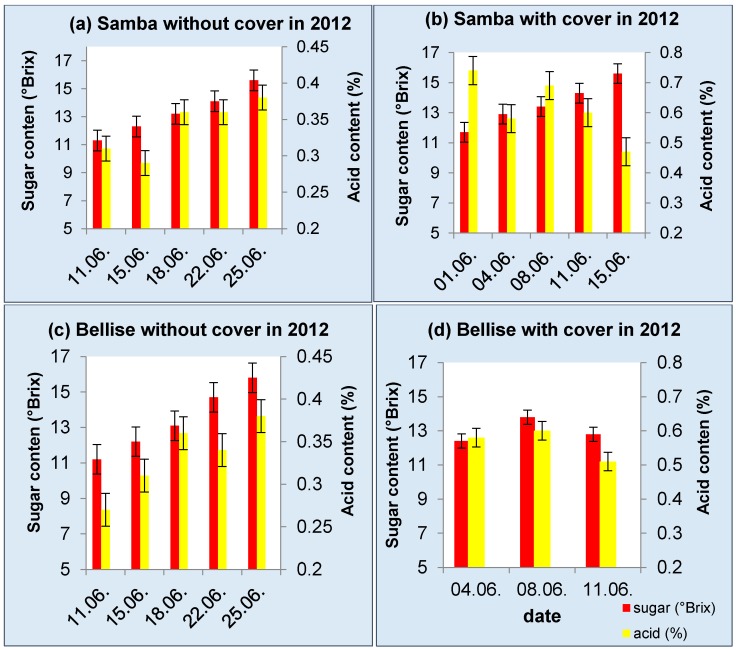
Sugar and acid content in 2012 of cv. “Samba” without (**a**) and with cover (**b**) and “Bellise” without (**c**) and with cover (**d**) (N.B. right-hand axis has different scaling, depending on treatment).

The sugar content increased concomitantly during maturation in all treatments from a minimum 11.2° Brix to a maximum of 15.6° Brix (Figure 2). The acid content *decreased* from 0.58% to 0.51% in “Bellise” with cover and from 0.74% to 0.47% in “Samba” with cover, but *increased* during ripening from 0.27% in “Bellise” and 0.31% in “Samba” to 0.38% in both treatments without cover (Figure 2); hence, the slow and minor changes in acid content do not appear to be a good maturity parameter.

### 3.3. Proposed Maturity Model

**Figure 3:**
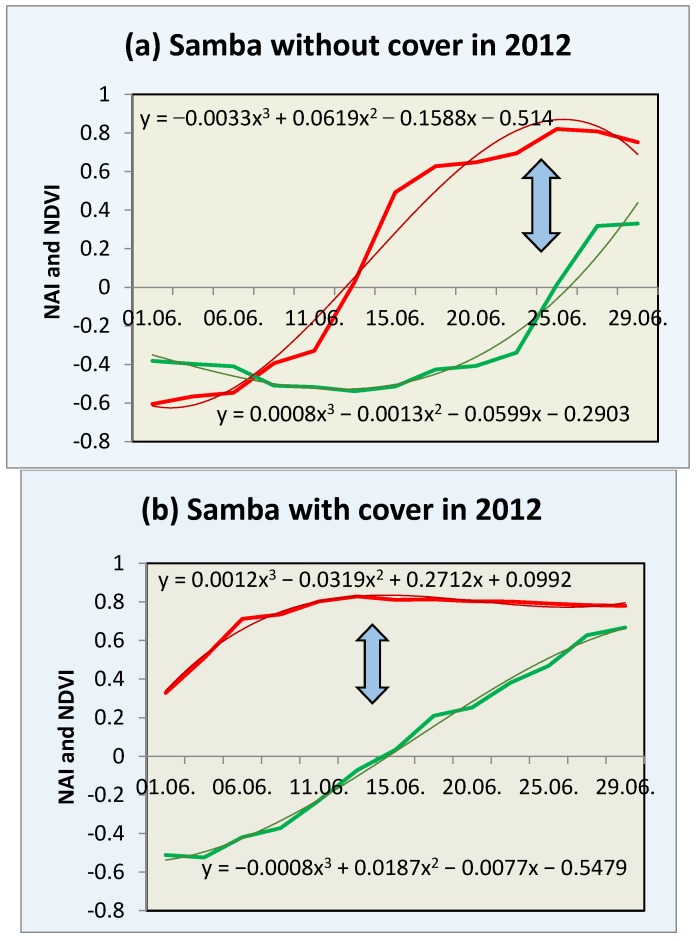

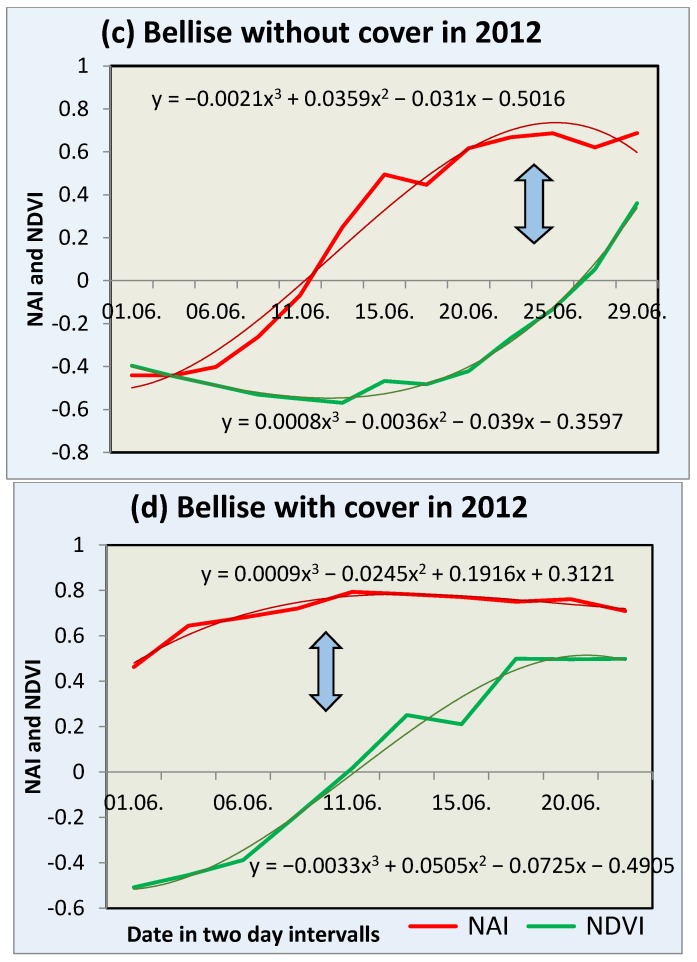
NAI and NDVI values in 2012 of cv. “Samba” without (**a**) and with cover (**b**) and “Bellise” without (**c**) and with cover (**d**). *n* = 30, polynomial function evaluate with median of the measurements; arrows show the optimum harvest (OHD).

The concentration of anthocyanin, the pigment responsible for the red coloration in cherry fruit, increased consistently with maturation in cv. “Samba” and “Bellise” in both years and in both cultivation systems (i.e., inside and outside the polytunnel) (Table 2 and Figure 3).

**Figure 4:**
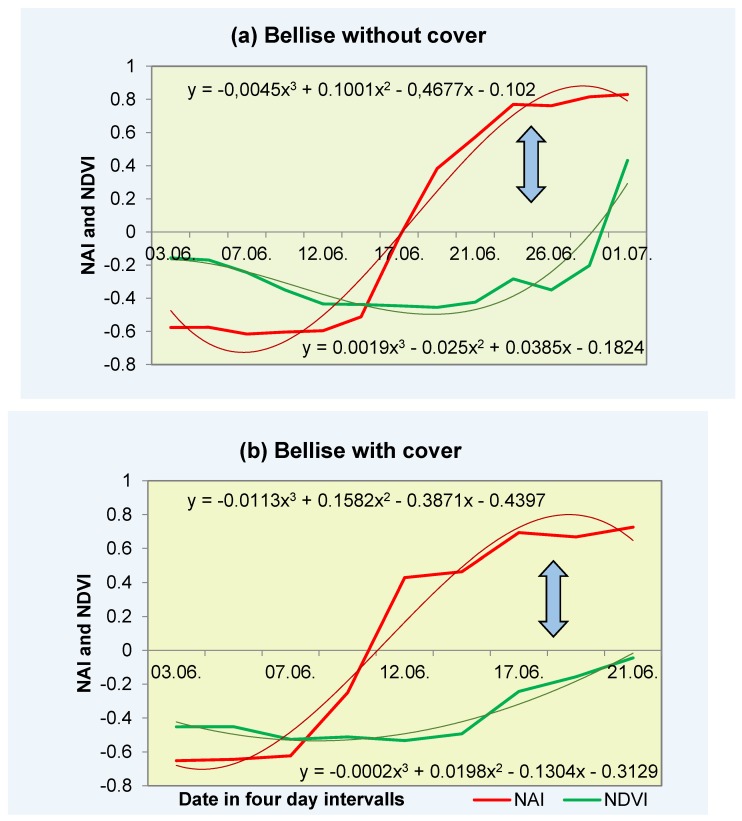
NAI and NDVI levels in 2013 of cv. “Bellise” without (**a**) and with cover (**b**); *n* = 20, polynomial function evaluate with median of the measurements; arrows show the optimum harvest date (OHD).

Sensor values of NAI, representative of the relative anthocyanin content in cherry, became less negative from ca. −0.6 relative units in the young, hard, and slightly yellow (breaker stage), concomitant with the onset of the visual appearance of red coloration, to a maximum of +0.8 in mature fruits. In 2012, fruit under cover were an exception, because NAI values showed advanced ripening with NAI values of +0.3 to +0.4 at the start of the measurements, but not in the open field. This results in a divergent graphical representation of the measurements for the fruit under cover, because the data did not include the onset of the maturation process (Figure 3b,d). Similarly, NDVI, representative of the relative chlorophyll content in the cherry fruit, concomitantly became less negative from −0.4 relative units in the fruits to +0.5 in overripe fruits (Figure 3 and Figure 4).

0 < NDVI (t) < 0.2, NDVI’(t) = 0(1)

0.7 < NAI (t) < 0.8, NAI’(t) = 0(2)

In the proposed model, the optimum harvest date (OHD) is reached when (a) NDVI = 0 (Equation (1)) and (b) the first derivative of the NAI function becomes zero (Equation (2)).

The criteria for the optimum harvest date in all cherry cultivars and cultivation systems examined was a relative anthocyanin level viz. NAI between +0.7 and +0.8 (Equation (2)) and a chlorophyll degradation, expressed as an NDVI between 0.0 and +0.2 (Equation (1)) (Figure 3 and Figure 4). In overripe fruits, the relative anthocyanin level plateaued at a maximum NAI value of 0.8.

### 3.4. Harvest Prediction

In the apparent absence of a maturity index for stone fruit (including cherry, plum, nectarine, apricot etc.), a model was developed to predict fruit maturation as closely as possible based on nondestructive chlorophyll and anthocyanin measurement with a portable, battery-driven pigment analyzer. Upon maturation, NAI values plateaued at +0.8 (Figure 3 and Figure 4) in the case of overripe fruits, resulting in a sigmoidal curve pattern when polynomial curve fitting is used. The first derivative of the NAI function (Equation (2)) (normalized anthocyanin content) approaches zero, indicating the harvest date (Figure 3 and Figure 4), while the NDVI (normalized chlorophyll index) exceeds zero (Figure 3 and Figure 4).

Proposed maturity formula for the OHD:

(3) OHD=t0 + (12 to 14) days

where, OHD—optimum harvest date, t0—date, when:

(4) NAIvalue =0 and −0.5 <NDVIvalue<−0.4

The optimum harvest date can vary ±2 days dependent on growth conditions (e.g., sunny days accelerate ripening and cloudy, cool and rainy conditions delayed ripening processes. For example, if the NAI is −0.55 and the NDVI is −0.4, the OHD will be after 19 days (Figure 3). The proposed maturity formula probably applies also to fruit under cover in 2012, if one extrapolates the data virtually.

The harvest was correlated with fruit quality parameters (e.g., sugar–acid relation, maturity, or fruit size) of each cherry cultivar. For example, fruit size and fruit weight increased similar to NAI and NDVI values during ripening (Table 1).

## 4. Discussion

The objective of the study was to develop an innovative maturity index using modern nondestructive sensor technology to determine and predict the optimum harvest date for sweet cherry. So far, stone fruit like cherry, plum, apricot, and nectarine lack a maturity index to predict the optimum harvest date [7]. In the case of peach, Grossman and DeJong [18] created a simulation model (PEACH) based on photosynthetic carbon assimilation and daily minimum and maximum temperature and solar radiation as inputs. Based on their model, Ben Mimoun and DeJong [19] developed the model futher and used the relationship between the accumulation of growing degree hours during 30 days after full bloom and the harvest date and the number of days between full bloom and harvest maturity to predict harvest date and yield for peach [20].

In the present experiment lasting 2 years with two sweet cherry cultivars—each having different ripening behaviors—and employing two different cultivation systems, a different approach (i.e., physiological rather than climatic) was used. Nondestructive measurements of the relative changes in pigment content during fruit ripening proved the most successful approach to determine the optimum harvest date of sweet cherry. The coincidence of the optimum ripening parameters such as firmness, sugar content, fruit size, and maximum anthocyanins content (analyzed chemically), and the NAI value (measured non-destructively by the sensor), appeared to be a suitable approach. Both NAI and NVDI reach a plateau at the optimum harvest time, making NAI a suitable candidate for the first derivative as it approaches zero (Equation (2)). In the two cherry cultivars, NAI values increased from −0.6 to +0.8 during maturation, while the NDVI became less negative, from −0.4 close to zero at the optimum harvest date. An equation was developed, when the NAI becomes zero and the NDVI is between −0.5 and −0.4, to predict the number of days until the optimum harvest date (Equation (3)).

Spectrometry in the visible wavelengths is a promising tool to determine pigment content during fruit ripening. Zude-Sasse et al. [12] showed for apple that chlorophyll degradation, measured by spectrometer arrays, is a sensitive indicator of physiological fruit ripening and influences consumer behaviour. Kuckenberg et al. [21] demonstrated also in the case of apple that fruit ground color alterations associated with chlorophyll breakdown can successfully be monitored by light remission techniques (e.g., with a pigment analyzer and with laser-induced fluorescence). The latter, however, proved unsuitable for stone fruits such as cherry because of the absence of chlorophyll at the end of the maturation (Blanke, 2011 unpublished). Blanke et al. [22] were the first ones to distinguish between varieties and ripening stage viz. red coloration in cherry using spectral (VIS) light reflection measurements with a portable, non-destructive unit (‘UNISPEC, PPSystems, Amesbury, MD, USA).

During fruit ripening, red color formation at first reflects higher flavonoid—particularly anthocyanin—content, and also improves the nutritional value of the fruit [23]. Epidemiological and intervention studies have provided evidence of beneficial health effects of dietary fruits and vegetables, and the beneficial effects have been attributed in part to secondary plant components, including flavonoids and other phenolic compounds [24]. Effects of flavonoids in reducing the risk of various diseases—including cardiovascular disease, cancer, atherosclerosis, and other age-related diseases—have been demonstrated [6,25].

In the present experiment, the pigment content of sweet cherry fruits was measured nondestructively with modern sensor technology (i.e., a portable pigment analyzer) during fruit maturation. The results show that the NAI and the NDVI increased during maturation in the same way as the other fruit quality parameters (e.g., fruit weight, size, and sugar). These results concur with the chemical analysis and show the highest amount of anthocyanins on the harvest date and thereafter (Table 2 and Figure 3 and Figure 4). Cherry fruits differ from pome fruits, such as apple, in that synthesized pigments are not only in the sun-exposed side of the fruit [14], but also in the inner shade-side of the fruit and throughout the flesh. The in situ measurements with the portable, battery-driven, affordable pigment analyzer are simple and quick and make elaborate and time-consuming chemical laboratory analysis redundant for the determination of the optimum harvest date. Instruments like the pigment analyzer compare favorably with colorimeters, because they evaluate two indices, which constitute the progress of ripening. Zude [26] described a decreasing NDVI related to a decreasing chlorophyll a content during maturation. The results showed that the spectral optical pigment determination is a promising tool to evaluate the development of ripeness [12]. To our knowledge, this plant sensor (pigment analyzer) was only used to evaluate the NAI and NDVI in banana, apple, and tomato fruit, but not stone fruit. Hence, the head of the pigment analyzer was too big for measurements on cherry fruits without modification, so we machined a matt black holder (Figure 1). In the future, a modified smaller head of the instrument according to the fruit size of cherries and other small fruits may be an option.

## 5. Conclusions

To our knowledge, this is the first model or index for a stone fruit based on both green (chlorophyll degradation) and red (anthocyanin) de novo biosynthesis, and the first derivative of NAI to describe maturation and predict the optimum harvest date for cherry fruit; future work will show which modifications are necessary to apply this idea to cherry and possibly other fruit, regional growing conditions, cultivation mode, year and cultivars employed.

## Figures and Tables

**Table:** (**a**)

Date	29.05	01.06	04.06	08.06	11.06	15.06
Bellise with cover						
Fruit size (mm); SD	22.0 ± 1.0	23.5 ± 1.8	22.9 ± 2.3	24.7 ± 2.0	25.6 ± 1.7	
Fruit weight (g); SD	5.6 ± 0.6	6.1 ± 1.2	6.3 ± 1.7	7.5 ± 1.6	8.4 ± 1.5	
Firmness (Shore A); SD	64.8 ± 14.8	65.9 ± 24.2	66.0 ± 25.7	52.7 ± 14.9	54.9 ± 21.3	
Samba with cover						
Fruit size (mm); SD	19.7 ± 0.7	21.6 ± 1.2	23.0 ± 1.6	23.8 ± 1.6	24.8 ± 1.6	25.8 ± 1.3
Fruit weight (g); SD	4.8 ± 0.4	5.6 ± 0.8	6.6 ± 1.2	7.6 ± 1.2	8.2 ± 1.3	9.6 ± 1.2
Firmness (Shore A); SD	86.7 ± 11.7	53.1 ± 28.8	77.9 ± 15.7	62.5 ± 18.6	69.3 ± 12.9	75.8 ± 11.5

**Table:** (**b**)

Date	29.05	01.06	04.06	08.06	11.06	15.06	18.06	22.06	25.06
Bellise without cover									
Fruit size (mm); SD	16.5 ± 1.1	18.5 ± 1.1	20.1 ± 1.3	20.3 ± 1.1	21.1 ± 1.2	22.6 ± 1.1	22.8 ± 1.4	23.9 ± 1.5	24.3 ± 1.4
Fruit weight (g); SD	2.6 ± 0.5	3.2 ± 0.5	4.0 ± 0.6	4.2 ± 0.6	4.8 ± 0.7	5.9 ± 0.8	6.2 ± 0.9	7.7 ± 1.3	7.9 ± 1.1
Firmness (Shore A); SD	100	100	100	100	100	100	100	75.8 ± 11.8	70.9 ± 12.4
Samba without cover									
Fruit size (mm); SD	17.2 ± 0.6	18.2 ± 1.1	19.1 ± 1.6	20.5 ± 1.5	20.9 ± 1.0	21.4 ± 1.4	21.9 ± 1.3	23.1 ± 1.2	24.5 ± 1.1
Fruit weight (g); SD	3.2 ± 0.2	3.6 ± 0.2	4.1 ± 0.7	4.9 ± 0.8	5.3 ± 0.7	6.0 ± 1.1	6.4 ± 1.0	7.5 ± 1.1	8.7 ± 1.1
Firmness (Shore A); SD	100	100	100	100	100	100	100	74.9 ± 7.5	70.5 ± 8.7

**Table:** (**a**)

Date	29.05	01.06	04.06	08.06	11.06	15.06	18.06
Bellise with cover							
Chl a (nmol·g^−1^)	0.07	0.06	0.06	0.01	0.01		
Chl b (nmol·g^−1^)	0.03	0.02	n.d.	0.02	n.d.		
Carotenoids (nmol·g^−1^)	1.39	1.93	0.71	0.02	1.35		
Flavonoids (nmol·g^−1^)	34.5	45.2	41.3	78.8	85.9		
Anthocyanins (nmol·g^−1^)	1.38	5.79	27.2	106.5	190.7		
Samba with cover							
Chl a (nmol·g^−1^)	0.26	0.33	1.14	0.06	0.15	0.03	0.00
Chl b (nmol·g^−1^)	0.03	0.10	1.7	0.03	0.33	0.11	n.d.
Carotenoids (nmol·g^−1^)	2.08	2.27	5.42	1.42	0.72	0.16	1.35
Flavonoids (nmol·g^−1^)	153	162	208	154	193	198	234
Anthocyanins (nmol·g^−1^)	7.43	11.96	7.97	11.1	45.3	69.6	106.6

**Table:** (**b**)

Date	29.05	01.06	04.06	08.06	11.06	15.06	18.06	22.06	25.06
Bellise without cover									
Chl a (nmol·g^−1^)	3.53	0.02	0.00	0.20	0.07	n.d.	n.d.	n.d.	n.d.
Chl b (nmol·g^−^)	2.16	0.07	n.d.	0.03	0.02	n.d.	n.d.	n.d.	n.d.
Carotenoids (nmol·g^−1^)	5.08	0.06	0.00	3.10	1.67	0.56	n.d.	n.d.	1.14
Flavonoids (nmol·g^−1^)	92.2	94.4	33.7	70.6	51.5	45.8	53.49	86.4	84.7
Anthocyanins (nmol·g^−1^)	5.58	7.27	3.65	14.33	4.46	13.07	11.28	30.3	114.3
Samba without cover									
Chl a (nmol·g^−1^)	0.01	0.00	0.05	0.25	n.d.	0.12	0.02	0.05	n.d.
Chl b (nmol·g^−1^)	0.01	n.d.	n.d.	0.05	n.d.	0.25	n.d.	0.07	n.d.
Carotenoids (nmol·g^−1^)	0.01	0.00	0.07	1.99	0.05	1.18	1.45	1.43	n.d.
Flavonoids (nmol·g^−1^)	188	242	255	175	171	178	176	169	215
Anthocyanins (nmol·g^−1^)	9.47	6.16	5.50	5.18	4.78	15.6	36.9	31.3	172.8

## Abbreviations

The following abbreviations are used in this manuscript:

NAI	Normalised anthocyanins index
NDVI	Normalised differential vegetation index
OHD	Optimum harvest date
